# Synthesis and structural characterization of a new dinuclear platinum(III) complex, [Pt_2_Cl_4_(NH_3_)_2_{μ-HN=C(O)Bu^t^}_2_], and Synthesis and structure of two novel *trans*-platinum complexes. Addenda and errata

**DOI:** 10.1107/S205252062400492X

**Published:** 2024-07-31

**Authors:** IUCr Editorial Office

**Affiliations:** a 5 Abbey Square, Chester CH1 2HU, England

**Keywords:** platinum complexes, anticancer drugs, X-ray diffraction, crystal structure, errata

## Abstract

This article reports changes to the authorship of two papers by Vinci & Chateigner [(2022). *Acta Cryst.* B**78**, 835–841; (2023). *Acta Cryst.* B**79**, 213–219], as well as providing additional literature citations.

## Abstract 2

Articles by Vinci & Chateigner [(2022). *Acta Cryst.* B**78**, 835–841; (2023). *Acta Cryst.* B**79**, 213–219] are corrected.

The author lists for two articles by Vinci & Chateigner (2022 ▸, 2023 ▸), should include the following additional authors: F. P. Intini (Department of Chemistry, University of Bari Aldo Moro, Italy), E. Mesto (Department of Earth and Geoenvironmental Sciences, University of Bari Aldo Moro, Italy), C. Pacifico (Department of Chemistry, University of Bari Aldo Moro, Italy) and E. Schingaro (Department of Earth and Geoenvironmental Sciences, University of Bari Aldo Moro, Italy). The acknowledgements to Professor Intini in both papers are therefore removed.

In Vinci & Chateigner (2022 ▸), a reference to the syntheses of [PtI_2_(NH_3_)(NCBu^*t*^)], [PtCl_2_(NH_3_)(NCBu^*t*^)] and [PtCl_4_(NH_3_)(NCBu^*t*^)] by Sinisi *et al.* (2014 ▸) is added. In Vinci & Chateigner (2023 ▸), a reference to the synthesis, NMR and chemical characterization of compound **1** and of its precursor by Cini *et al.* (1993 ▸) is included.

In Table 1 of Vinci & Chateigner (2022 ▸), for the analysed compound, the absorption correction yielded *T*_min_ = 0.347, *T*_max_ = 0.747, and the number of reflections with *I* > 2σ should be 4938 and not 4944.

In Vinci & Chateigner (2023 ▸), an erroneous formula is given in Section 2.1 for the saline precursor in the synthesis. The correct formula is K_2_[PtCl_2_{(HNC(O)Ph)}_2_]. In the same paper, in Table 1 for compound **1**, *T*_min_ = 0.544, *T*_max_ = 0.748, and the number of reflections with *I* > 2σ should be 3371 and not 3466. The dimensions of the investigated crystal of compound **2** are 0.040 × 0.122 × 0.200 mm, the absorption correction yielded *T*_min_ = 0.412, *T*_max_ = 0.748, and the number of reflections with *I* > 2σ should be 5527 and not 5194.

The above corrections have been made with the agreement of all authors concerned, the Section Editors of *Acta Crystallographica B* and the Editor-in-chief of IUCr Journals.
